# Systematic Review and Meta-Analysis of Artemisinin Based Therapies for the Treatment and Prevention of Schistosomiasis

**DOI:** 10.1371/journal.pone.0045867

**Published:** 2012-09-21

**Authors:** Luis Pérez del Villar, Francisco J. Burguillo, Julio López-Abán, Antonio Muro

**Affiliations:** 1 Laboratorio de Inmunología y Parasitología Molecular, IBSAL-CIETUS, Facultad de Farmacia, Universidad de Salamanca, Salamanca, Spain; 2 Departamento Química Física, Facultad de Farmacia, Universidad de Salamanca, Salamanca, Spain; Swiss Tropical and Public Health Institute, Switzerland

## Abstract

**Background:**

Chemotherapy based on repeated doses of praziquantel is still the most effective control strategy against Schistosomiasis, however artemisinin derivatives emerged as a family of compounds with schistomicide activity. The aim of the present work is to compare the efficacy of artemisinin-based therapies in the treatment and prophylaxis of human schistosomiasis. The design of this work involved a quantitative systematic review and meta-analysis.

**Methodology/Principal Findings:**

Retrieval of published studies was carried out through an electronic search of the PubMed (MEDLINE), EMBASE, Cochrane Library and CINAHL databases. This included reports comparing the therapeutic efficacy of artesunate alone, artesunate *plus* sulfadoxine-pyrimethamine and a combination of artemisinin derivatives *plus* praziquantel against praziquantel alone on different types of schistosomiasis. Moreover, studies on artesunate and artemether used as preventive drugs were also analyzed against placebo. The primary outcome measure for schistosomiasis treatment was “parasitological cure”, whereas for the prophylaxis the outcome evaluated was “infection rate”. Our results show that patients treated with artesunate alone have significantly lower cure rates than those treated with praziquantel (OR = 0.27 (95% C.I. 0.13–0.53; p<0.001)) and that the combined therapy of artesunate *plus* sulfadoxine-pyrimethamine is also significantly less effective than praziquantel treatment (OR = 0.14 (95% C.I. 0.02–0.92; p = 0.04)). However, the combination of an artemisinin derivatives *plus* praziquantel showed a higher cure rate than praziquantel monotherapy with OR = 2.07 (95% C.I. 1.27–3.36; p = 0.003). Finally, chemoprophylaxis with either artesunate (RR = 0.11 (95% C.I. 0.06–0.22; p<0.001)) or artemether (RR = 0.25 (95% C.I. 0.16–0.40; p<0.001)) was significantly better than a placebo in both cases.

**Conclusions/Significance:**

This meta-analysis confirms that artemisinin derivatives used in combination with praziquantel have the potential to increase the cure rates in schistosomiasis treatment, but not artesunate alone. It is also confirmed that repeated doses of artemisinin derivatives play a prophylactic role, significantly reducing the incidence of *Schistosoma japonicum* infections compared with placebo.

## Introduction

Schistosomiasis continues to be one of the most prevalent parasitic diseases. An estimated 779 million people - more than 10% of the world’s population - were at risk of schistosomiasis in mid-2003 and approximately 207 million people were infected [1]. The causative agent of schistosomiasis is a trematode worm of the genus *Schistosoma*. Three main species parasitize humans: *S. haematobium*, *S. japonicum*, and *S. mansoni*. Additionally, *S. intercalatum* arises in some parts of Central Africa and *S. mekongi* as well as *S. malayensis* occur in Southeast Asia [2]–[4]. An alarming 201.5 million cases of schistosome infections (mainly by *S. haematobium* and *S. mansoni*) have been estimated to occur in Africa, accounting for more than 97% of the estimated number of infections worldwide [5]. The highest prevalence of this illness is usually found in school-age children and adolescents, where it represents the main cause of iron deficiency anaemia [6]. The World Health Organization (WHO) reports the effect of schistosomiasis on world health as the loss of 1.7 and 4.5 million disability-adjusted life years [7], [8]. However, a recent systematic review has shown that schistosomiasis-related disability is underestimated [9]. The treatment of this chronic and debilitating disease relies on the use of praziquantel, a broad-spectrum schistosomicide drug that combines safety and low price [10]. Praziquantel is active against the adult stages of schistosomes. Thus, the main limitation to the use of praziquantel is the lack of therapeutic efficacy against early-stage schistosomiasis which could be the main reason for the many treatment failures observed and high rates of re-infection [11], [12]. Moreover, current control programmes against schistosomiasis depend on the wide-scale use of praziquantel. Consequently, this drug pressure could favor the emergence of praziquantel-resistant parasites [11]. Thus, it is not appropriate to rely on a single drug regimen for the control of schistosomiasis.

The antischistosomal activity of the artemisinin derivatives was discovered in the early 1980’s leading this family of compounds to become used as broad-spectrum antischistosomal drugs [13]. Artemisinin derivatives act against the developmental stages of the parasite and are therefore complementary to praziquantel activity [14]. The incorporation of artemisinin derivatives into schistosomiasis treatment in combination with praziquantel may therefore represent a good strategy. However, several reviews have suggested that artemisinin derivatives exert only a moderate degree of efficacy against schistosomiasis [7], [14], [15]. Meanwhile, artemisinin derivatives have been used in successfully combination with other compounds for the treatment of malaria, reducing the probability of selecting drug-resistant parasites [16], [17]. Moreover, synthetic artemisins (e.g. trioxolanes) are also active against juvenile and adult schistosomes [18], but no schistosome-specific clinical trials with synthetic artemisinins have been undertaken so far.

The aim of the present systematic review is to analyse the data available from clinical trials that compare the efficacy of artemisinin derivatives for the treatment and prophylaxis of schistosomiasis. We hope to provide clinicians and policy-makers with a convenient and evidence-based summary of the primary literature on which to base their decisions.

## Methods

### Searching

We carried out a systematic search of published studies of clinical trials that compare anti-helminthic therapies and chemoprophylaxis based on artemisinin derivatives against schistosomiasis, with no date or language restrictions. We used PubMed (MEDLINE), EMBASE, the Cochrane Library and CINAHL, up to July 2012. We incorporated the following search terms: “schistosomiasis”, “praziquantel”, “artesunate”, “artemether” and “artemisinin derivatives”. A customized form was used to record the name of the authors and journal, the year of publication, the location of the trial, the intensity of infection, the study design, the inclusion criteria, dosage, population characteristics, and outcomes.

### Inclusion Criteria and Outcomes

Published reports evaluating artemisinin derivatives in schistosomiasis treatment were included if they fulfilled all of the following selection criteria according to PRISMA guidelines [19]. **(1) Population:** Patients infected with *S. haematobium, S mansoni* and *S. japonicum* diagnosed through Kato-Katz thick smears (*S. mansoni* and *S. japonicum*) or filtration of 10 ml of urine (*S. haematobium*). *S. intercalatum*, *S. mekongi* and *S. malayensis* were not considered since there were no clinical trials focused in these species. **(2) Study design and interventions:** We included randomized trials comparing the efficacy of artemisinin-based therapies against praziquantel alone, the reference treatment option for all types of schistosomiasis. **(3) Types of outcomes measures:** As the main outcome, we considered “parasitological cure”, defined as the absence of eggs over a short period of time (3 to 8 weeks after treatment). Furthermore, adverse events were defined as secondary outcome to be considered in the systematic review.

To evaluate schistosomiasis chemoprophylaxis based on artemisinin derivatives, we included articles if they fulfilled all of the following selection criteria. **(1) Population:** healthy villagers that lived in endemic areas of schistosomiaisis. **(2) Study design and interventions:** we included randomized clinical trials, comparing the prophylactic effect of artesunate or artemether *vs.* placebo against *S. haematobium, S. mansoni* and *S. japonicum* infections. The preventive effect of the artemisinin derivatives was assessed by Kato-Katz thick smears or by the filtration of 10 mL of urine for *S. japonicum* and *S. haematobium,* respectively. **(3) Types of outcomes measures:** the main outcome was measured in terms of “infection rate”, defined as number of patients infected with *Schistosoma spp.* against total number of patients included in any branch of the study, over a short period of time (3 to 4 weeks after treatment). Adverse events were also defined as secondary outcome measure.

### Data Extraction and Synthesis

Two authors (L.P.V. and J.L.A.) independently extracted the data using a data extraction sheet designed by authors. Extraction sheets for each study were crosschecked for consistency, and any discrepancies resolved by discussion. Disagreements between reviewers were resolved by consensus or input from a third author (A.M.).

### Quality Assesment within Individual Studies

We performed an evaluation of the risk of bias for each publication based on four elements of study design and reporting: (1) a description of randomization method and masking of patient and practitioner; (2) a description of allocation sequence generation; (3) reported allocation concealment, masking of participants, study personnel and outcome assessors; (4) reported avoidance of incomplete outcome data (dropouts/withdrawals). A score of 4 was considered high quality, 2–3 moderate quality, 0–1 poor quality according to a modified Jadad score [20]. We also evaluated any additional information related with inclusion and exclusion criteria like sample size calculation, baseline comparability of age, gender and relevant clinical characteristics (follow up and diagnoses).

### Quantitative Data Synthesis

To compare efficacy of schistosomiasis treatments, the outcome measure was expressed as the odds ratio (OR) between the alternative treatment and the control, together with its 95% confidence interval (95% C.I.). Regarding studies that have evaluated the chemoprophylaxis activity of artemisinin derivatives in schistosomiasis, we used “infection rate”, instead of “cure rate”, and the data were referred to relative risk (RR) instead of OR since the RR index is more informative in terms of prevention studies. All p-values reported for OR and RR were calculated using a Z-test for the null hypothesis (i.e. OR = 1 and RR = 1) [21].

To assess the heterogeneity between studies a Cochran’s Chi-square test (Q-test) was made under the null hypothesis of homogeneity (significant heterogeneity if p<0.05). Additionally, the I^2^ statistic was calculated and values greater than 50% were considered as high heterogeneity. The fixed-effect model, using the inverse variance method, was used where there was no evidence of heterogeneity and a common effect size could be assumed. However, the random-effects model was chosen when heterogeneity was detected and the true effect size varied between studies. To estimate the between studies variance (*tau* squared) the DerSimonian and Laird approach was used [22].

When several groups (species of parasites) were included in a comparison, a subgroup analysis was performed. In the fixed-effect case, a fixed-effect model was used within subgroups and a fixed-effect summary was also calculated ignoring subgroups memebership. In the random-effects model, a meta-analysis was performed over to combine studies within each subgroup using separate estimates of tau-squared. Then the mean effects of subgroups were compared by the Q test to analyse if the difference was significant. Finnally, a global combined effect across subgroups was calculated by a separate random-effect meta-analysis including all the studies and ignoring subgroups membership since this option may be the more logical [21].

To assess the presence of publication bias, a funnel plot of effect size against standard error was analysed for each meta-analysis. Complementary to the funnel plot, the Egger’s regression asymmetry test was performed to quantify significance of publication bias [23]. All the above statistical analyses and graphs were performed with meta R-package [24].

## Results

### Study Selection

**Figure 1:**
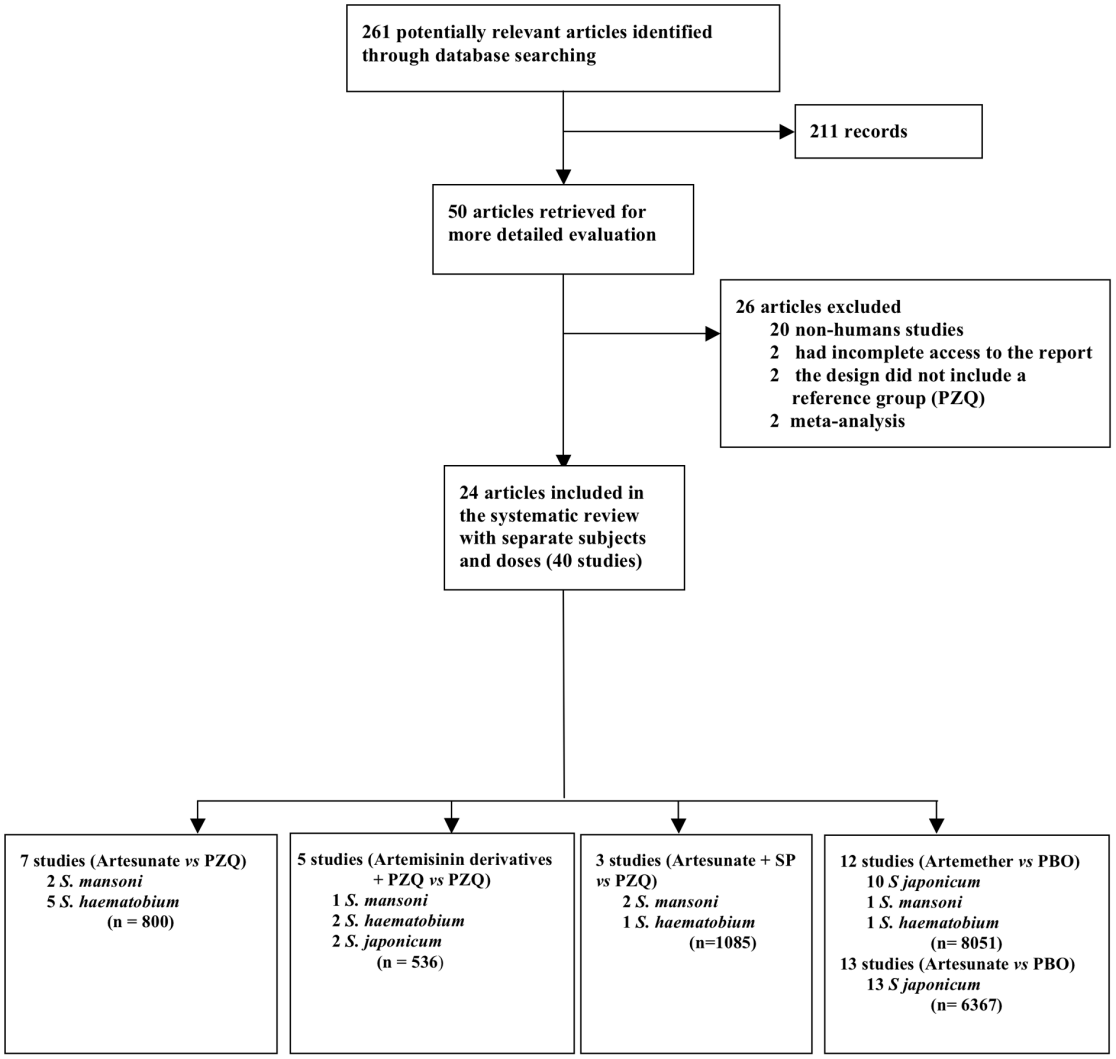
Flow diagram showing the selection process of studies included in the meta-analysis.

Reports from 261 published articles were screened for evidence of clinical trials that evaluated the efficacy of artemisinin derivatives in schistosomiasis treatment or prophylaxis. Of them, 50 articles were selected for more detailed evaluation and finally twenty-four published articles were included in the meta-analysis. Some published reports included in the meta-analysis are multicenter or evaluted the intervention with separate doses and protocols of treatment and they were included as different studies. A study is therefore considered as the main unit of the meta-analysis (see tables 1,2,3,4); The study selection process is outlined in Figure 1. When different endpoints were assessed in the same subjects we only considered the value reported for the first period of time of the follow up, since the other endpoints using the same subjects were not truly independent trials and data from a trial can only appear once in a specific analysis. Details of the included studies focused on the treatment and prophylaxis of schistosomiasis are given in table S1 and table S2, respectively. In addition, table S3 shows the 27 checklist items pertain to the content of a systematic review and meta-analysis according to PRISMA guidelines [19].

**Table 1:** Artesunate monotherapy *vs.* praziquantel in schistosomiasis treatment.

Citation	Year (trial)	Parasite	Location (Country)	Study Population (age)	Time point analysed (weeks)	Treatment	N°Cured/N° Treated
Borrmann *et al*. 2001 [25]	2000	*S. haematobium*	Moyen-Ogooué Province (Gabon)	Children (5–13)	8	ART^1^	24/89
						PZQ^2^	65/89
De Clercq *et al*. 2002 (study 1) [26]	2000	*S. haematobium*	Lampsar village (Senegal)	Children (7–14)	5	ART^3^	18/90
						PZQ^2^	26/88
De Clercq *et al*. 2002 (study 2) [26]	2000	*S. haematobium*	Makhana village (Senegal)	Children (7–14)	5	ART^3^	21/44
						PZQ^2^	34/45
Inyang-Etoh *et al*. 2009 [27]	2005	*S. haematobium*	Adim village (Nigeria)	Children (4–20)	8	ART^1^	33/44
						PZQ^2^	31/42
Keiser *et al*. 2010 [28]	2008	*S. haematobium*	Guéssiguié (Côte dIvore)	Children (8–16)	4	ART^1^	5/20
						PZQ^2^	23/26
De Clercq *et al*. 2000 [29]	1999	*S. mansoni*	L. T. Salane village (Senegal)	Adults (n.d.)	5	ART^1^	8/35
						PZQ^2^	16/36
De Clercq *et al*., 2000b [30]	1998	*S. mansoni*	Richard Toll village (Senegal)	Adults (6–61)	5	ART^1^	35/114
						PZQ^2^	29/38

1 ART (artesunate 4 mg/kg) daily for three consecutive days*;*

2 PZQ (praziquantel 40 mg/kg once);

3 ART (artesunate 8 tablets of 50 mg over 5 days; i.e. 3,2,1,1,1); n.d: data not described.

**Table 2:** Artemisinin derivatives + praziquantel *vs.* praziquantel in the treatment of schistosomiasis.

Citation	Year (trial)	Parasite	Drug	Location (Country)	Study Population (age)	Time point analysed (weeks)	Treatment	N°Cured/N° Treated
Borrmann *et al*., 2001 [25]	2000	*S. haematobium*	Artesunate	Moyen-Ogooué Province (Gabon)	Children (5–13)	8	PZQ^1^+ART^2^	71/88
							PZQ^1^	65/89
Inyang-Etoh *et al*. 2009 [27]	2005	*S. haematobium*	Artesunate	Adim Village (Nigeria)	Children (4–20)	8	PZQ^1^+ART^3^	39/44
							PZQ^1^	32/44
De Clercq *et al*., 2000 [29]	1999	*S. mansoni*	Artesunate	L. T. Salane village (Senegal)	Adults (n.d.)	5	PZQ^1^+ART^4^	27/39
							PZQ^1^	16/36
Hou *et al*., 2008 (study 1) [31]	2003	*S. japonicum*	Artemether	Hunan Province (China)	Adults (10–60)	6	PZQ^5^+ART^6^	50/51
							PZQ^5^	53/55
Hou *et al*., 2008 (study 2) [31]	2003	*S. japonicum*	Artemether	Hunan Province (China)	Adults (10–60)	6	PZQ^7^+ART^6^	43/44
							PZQ^7^	44/46

1 PZQ (praziquantel 40 mg/kg once);

2 ART (artesunate 4 mg/kg/day, given once per day over 3 days);

3 ART (artesunate 4 mg/kg) daily for three consecutive days;

4 ART (artesunate 200mg) on the first day and (100 mg) daily for a further 4 days;

5 PZQ (praziquantel 1 day, 3×20 mg/kg);

6 ART (artemether 6 mg/kg once);^ 7^PZQ (praziquantel 6 days, 3×20 mg/kg). n.d: data not described.

**Table 3:** Artesunate + sulfadoxine-pyrimethamine *vs.* praziquantel in the treatment of schistosomiasis.

Citation	Year(trial)	Parasite	Location (Country)	Study Population (age)	Time point analysed (weeks)	Treatment	N°Cured/N° Treated
Sissoko *et al*., 2009 [32]	2007	*S. haematobium*	Bamako (Mali)	Children (6–15)	4	ART+SP^1^	172/392
						PZQ^2^	206/389
Mohamed *et al*., 2009 [51]	2008	*S. mansoni*	New Halfa (Sudan)	Children (8–17)	4	ART+SP^3^	27/46
						PZQ^2^	46/46
Obonyo *et al*., 2010 [33]	2009	*S. mansoni*	Rarieda (Kenya)	Children (6–15)	4	ART+ SP^4^	15/106
						PZQ^2^	69/106

1 ART: (artesunate 100 mg/day) + S (sulfadoxine 250 mg/day) + P (pyrimethamine 12.5 mg/day) over three days;

2 PZQ (praziquantel 40 mg/kg once);

3 ART (artesunate 4 mg/kg/day over three days) + SP (sulfadoxine-pyrimethamine 25mg/kg );

4 ART(artesunate 4 mg/kg/day over three days) + S (sulfadoxine 25mg/kg) + P (pyrimethamine 12.5 mg/day).

**Table 4:** Artesunate prophylaxis trials focused on *Schistosoma japonicum* infections in endemic areas of China.

Citation	Year (trial)	Parasite	Location (Country)	Study Population(age)	Time point analysed (weeks)	N° of doses(interval in weeks)	Treatment	N° Positive/N° Treated
Wu *et al*., 1995 [34]	1993	*S. japonicum*	Jiangxi province	Residents (5–60)	4	8 (1)	ART^1^	0/346
							PBO	15/323
Xu *et al*., 1999 (study 1) [35]	1997	*S. japonicum*	Jiangxi province	Residents (6–65)	4	4 (2)	ART^1^	2/273
							PBO	11/289
Xu *et al*., 1999 (study 2) [35]	1997	*S. japonicum*	Jiangxi province	Residents (6–65)	4	4 (2)	ART^1^	0/107
							PBO	7/111
Li *et al*., 1999 [36]	1997	*S. japonicum*	Jiangxi province	Residents (5–60)	4	12 (2)	ART^1^	0/43
							PBO	4/58
Zhang *et al*., 2000 (study 1) [37]	1993	*S. japonicum*	Anhui province	Residents (6–65)	4	6 (2)	ART^1^	2/380
							PBO	18/400
Zhang *et al*., 2000 (study 2) [37]	1993	*S. japonicum*	Anhui province	Residents (6–65)	4	12 (2)	ART^1^	1/323
							PBO	31/323
Zhang *et al*., 2000 (study 3) [37]	1993	*S. japonicum*	Hubei province	Residents (6–65)	4	3 (1)	ART^1^	2/168
							PBO	22/200
Zhang *et al*., 2000 (study 4) [37]	1993	*S. japonicum*	Jiangxi province	Residents (6–65)	4	8 (1)	ART^1^	0/467
							PBO	41/397
Zhang *et al*., 2000 (study 5) [37]	1993	*S. japonicum*	Jiangxi province	Residents (6–65)	4	5 (2)	ART^1^	5/283
							PBO	20/304
Zhang *et al*., 2000 (study 6) [37]	1993	*S. japonicum*	Jiangxi province	Residents (6–65)	4	3 (2)	ART^1^	6/51
							PBO	12/64
Lu *et al*., 2000 (study 1) [38]	1999	*S. japonicum*	Anhui province	Residents (5–60)	4	3 (2)	ART^1^	0/210
							PBO	13/208
Lu *et al*., 2000 (study 2) [38]	1999	*S. japonicum*	Anhui province	Residents (5–60)	4	5 (2)	ART^1^	0/311
							PBO	17/312
Lu *et al*., 2000 (study 3) [38]	1999	*S. japonicum*	Anhui province	Residents (5–60)	4	13 (2)	ART^1^	1/209
							PBO	18/207

1 ART: artesunate (6 mg/kg); PBO: placebo.

### Characteristics and Methodological Quality

Regarding schistosomiasis treatment using artemisinin based therapies, we defined three units of analysis: (1) artesunate in monotherapy *vs.* praziquantel (seven trials described in six published studies); (2) artemisinin derivative *plus* praziquantel *vs.* praziquantel alone (five trials described in four published studies); (3) artesunate *plus* sulfadoxine-pyrimethamine *vs.* praziquantel (three published studies). All published studies were performed in Africa [25]–[30], except one study focused on *S. japonicum*, which was performed in China [31]. There were no studies that examined the efficacy of artemisinin derivatives in the American continent. The pediatric population was the target of trials focused on *S. haematobium,* whereas the studies on *S. mansoni* and *S. japonicum* focused on adult populations. In all studies both sexes were enrolled and patients were followed up over a short period of time (4 to 24 weeks). The studies on schistosomiasis treatment varied in sample size from 83 to 800 participants. In general, the quality of the reporting and design of the clinical trials that evaluated the artemisinin derivatives in the treatment of schistosomiasis was medium (see supplementary table S1). One study was an open labelled exploratory trial [28] and five were randomized controlled clinical trials with an adequate generation of allocation sequence [25], [30]–[33]. The majority of studies provide a sample size framework and a scientific rationale for the sample size determination. The remaining four studies were scored as having a high risk of bias mainly because of inadequate methods to generate the sequence of randomization (alternation), lack of allocation concealment, and/or incomplete outcome data.

Respecting artemisinin derivatives in the prophylaxis of schistosomiasis, we performed the meta-analysis in two main units according to the artemisinin derivative used: (1) artesunate *vs.* placebo and (2) artemether *vs.* placebo. The chemoprophylaxis doses for artesunate and artemether were 6 mg/kg at 1 or 2 week intervals for up to 13 doses during the high transmision period (two to six months). In both cases, all patients enrolled in the trials were initially treated with praziquantel prior to random assignment. In all trials, the infection rate was the outcome measure, and the RR, including 95% C.I. was estimated. Tables 4 and 5 summarize the key features of the prophylactic effect of artesunate and artemether, respectively. With regard to artesunate, a group of 5 published reports (including 13 different studies) were suitable for the meta-analysis. These studies were performed in different endemic areas of schistosomiasis in China investigated the efficacy of artesunate as a chemoprophylactic agent against *S. japonicum* infections [34]–[38]. These reports were published in Chinese language with abstracts and tables in English but some translation was required. The design of these studies were randomized double blind. Some of them were multicenter including different doses and intervals of treatment [35], [37], [38]. The retrieved studies that included artesunate as chemoprophylactic drug in schistosomiasis japonica reported insufficient information (see supplementary table S2). In fact, the quality score range from 0 to 2. Furthermore, it was identified two additional studies Li *et al.* 1996 [39] and Tian *et al.* 2001 [40] but access to the full publicactions were not possible and they were excluded from the meta-analysis. No published reports evaluated artesunate prophylaxis for *S. mansoni* and *S. haematobium* infections. In respect of artemether, 10 articles (including 12 different studies) were considered eligible for the meta-analysis. Seven of those articles were dealing with *S. japonicum* and were published in Chinese language [41]–[47] and the other 3 articles focused on *S. japonicum*, *S. haematobium* and *S. mansoni* were published in English language [48]–[50]. Seven articles of the above studies were carried out between 1996 and 2006 in Jiangxi, Anhui, Hunan, and Yunnan provinces of China including individuals aged between 5 and 60 years who were in frequent contact with infested water [41]–[44], [46]–[48]. One additional report was conducted among flood-relief workers aged between 18–40 in Jiangxi province [45]. Finally, 2 further reports focused on *S. haematobium* and *S. mansoni* infections were developed in Africa [49], [50]. With regard to the quality of the artemether reports, the score values range from 1 to 3 (table S2). Thus, the quality of studies was higher than those retrieved studies focused on artesunate prophylaxis.

**Table 5:** Artemether prophylaxis *vs.* placebo in schistosomiasis due to *S. haematobium*, *S. mansoni* and *S. japonicum*.

Citation	Year (trial)	Parasite	Location (Country)	Study Population (age)	Time point analysed (weeks)	N° of doses (Interval in weeks)	Treatment	N° Positive/N° Treated
Goran *et al*., 2001 [49]	2000	*S. haematobium*	Taabo village (Côte d’Ivore)	Children (5–15)	3	6 (4)	ART^1^	76/156
							PBO	97/150
Utzinger *et al*., 2000 [50]	1998	*S. mansoni*	Fagnampleu village (Côte d’Ivore)	Schoolchildren (n.d.)	3	6 (3)	ART^1^	31/128
							PBO	68/140
Xiao *et al*., 1995 [41]	1994	*S. japonicum*	Hunan province (China)	Residents (4–65)	4	3 (2)	ART^1^	20/365
							PBO	51/376
Xiao *et al*., 1996 [42]	1995	*S. japonicum*	Yunnan province (China)	Residents (4–65)	4	4(2)	ART^1^	13/307
							PBO	46/306
Xu *et al*., 1997 [43]	1996	*S. japonicum*	Anhui province (China)	Residents (6–65)	4	11(2)	ART^1^	0/433
							PBO	40/452
Tian *et al*., 1997 [44]	1996	*S. japonicum*	Hunan province (China)	Residents (5–60)	4	10 (2)	ART^1^	5/290
							PBO	82/305
Song *et al*., 1998 (study 1) [45]	1996	*S. japonicum*	Jiangxi province (China)	Flood relief workers (18–40)	6	3 (2)	ART^1^	4/99
							PBO	44/110
Song *et al*., 1998 (study 2) [45]	1996	*S. japonicum*	Jiangxi province (China)	Flood relief workers (18–40)	6	2 (2)	ART^1^	0/103
							PBO	4/102
Wang *et al*., 1997 [46]	1997	*S. japonicum*	Yunnan province (China)	Residents (3–60)	4	10 (2)	ART^1^	23/789
							PBO	87/717
Li *et al*., 2005 [48]	2004	*S. japonicum*	Jiangxi province (China)	Residents (6–60)	4	11(2)	ART^1^	3/373
							PBO	56/361
Song *et al*., 2006 (study 1) [47]	2004	*S. japonicum*	Jiangtxi province (China)	Residents (6–65)	6–8	7 (4)	ART^1^	42/402
							PBO	79/587
Song *et al*., 2006 (study 2) [47]	2004	*S. japonicum*	Jiangtxi province (China)	Residents (6–65)	6–8	13 (2)	ART^1^PBO	14/41379/587

1 ART: artemether (6 mg/kg); n.d: data not described.

### Meta-analysis

#### Artesunate in monotherapy vs. praziquantel

**Figure 2:**
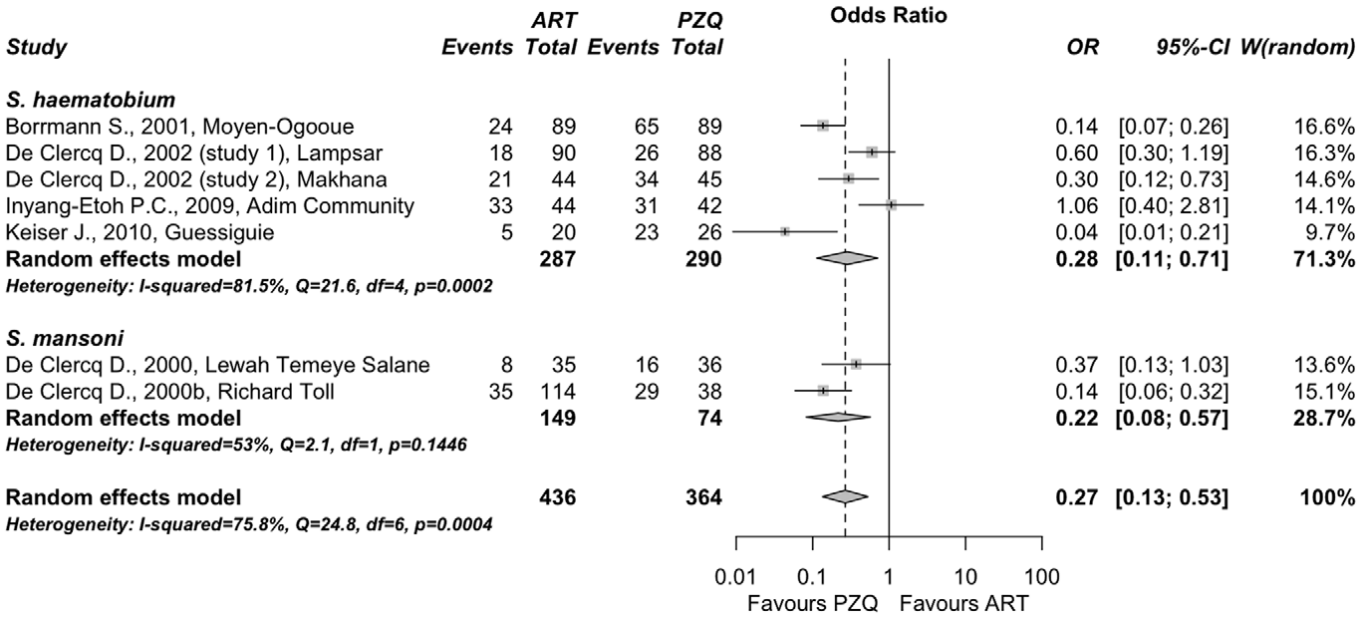
Forest plot of a random-effects subgroup meta-analysis comparing parasitological cure by artesunate as monotherapy *vs.* praziquantel. Points represent odds ratios with their corresponding 95% C.I. Intermediate diamonds are combined odds ratios for each subgroup and the diamond at the bottom is the overall combined odds ratio. The vertical line emphasizes an odds ratio = 1 (no difference) and the dashed vertical line shows the value of the overall combined odds ratio. The original reports are labeled with author name, year and location (for details see table 1).

A total of 7 studies (n = 800) included in six published reports [25]–[30] compared the efficacy of artesunate alone *vs.* praziquantel alone in sub-Saharan Africa. Four studies performed on populations of schoolchildren evaluated artesunate alone in *S. haematobium* infections, and two additional studies on adult populations assessed the efficacy of oral artesunate for the treatment of *S. mansoni*. Regarding to the doses of artesunate used, it must be noted that five reports used artesunate 4 mg/kg daily for three consecutive days; one additional report used eight tablets of artesunate 50 mg over five days; (i.e. 3,2,1,1,1). All these details were described in table 1. None of the studies evaluated the efficacy of artesunate on *S. japonicum* infection. There was significant heterogeneity in the effect size among all the studies (Q = 24.8 (p<0.001), I^2^ = 75.8% (>50%)). We therefore performed a subgroup random-effects meta-analysis (Figure 2). The results revealed that patients treated with artesunate alone had a significantly lower cure rate than those treated with praziquantel, both with *S. haematobium,* showing an OR = 0.28 (95% C.I. 0.11−0.71; p = 0.007), and with *S. mansoni* infections, giving an OR = 0.22 (95% C.I. 0.08−0.57; p = 0.002). Subgroup difference using the Q test was no significant (p = 0.69). Overall, the combined OR was 0.27 (95% C.I. 0.13−0.53; p<0.001), clearly showing that at least from the studies reviewed artesunate alone is significantly less effective than praziquantel treatment.

#### Artemisinin derivative plus praziquantel vs. praziquantel alone

**Figure 3:**
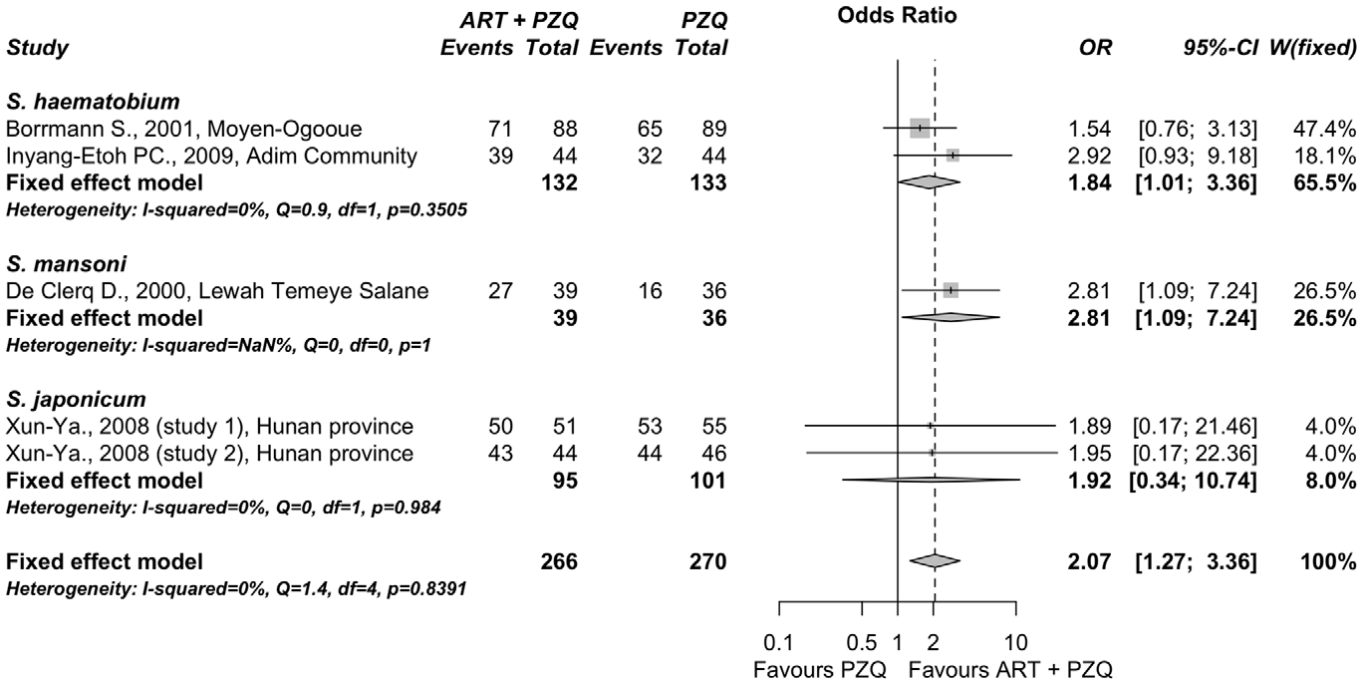
Forest plot of fixed-effect subgroup meta-analysis comparing parasitological cure after treatment with an artemisinin derivative *plus* praziquantel *vs.* praziquantel. Points represent odds ratios with their corresponding 95% C.I. Intermediate diamonds are combined odds ratios for each subgroup and the diamond at the bottom is the overall combined odds ratio. The vertical line emphasizes an odds ratio = 1 (no difference) and the dashed vertical line shows the value of the overall combined odds ratio. The original reports are labeled with author name, year and location (for details see table 2).

With respect to combination therapies, four published reports [25], [27], [29], [31] described five studies (n = 536) in which the effectiveness of artemisinin derivatives in combination with praziquantel was compared against praziquantel alone. This comparison included two studies involving *S. haematobium* and a single study with *S. mansoni,* all using artesunate as the drug. Furthermore, two additional studies for *S. japonicum* were incorporated in which artemether was used instead of artesunate. The different dosages used for combination therapies were described in table 2. No significant heterogeneity was identified among all of the studies (Q = 1.43 (p = 0.839), I^2^ = 0% (<50%)). According to that, a subgroup fixed-effect meta-analysis was performed (Figure 3). Patients treated with artesunate *plus* praziquantel in *S. haematobium* infections showed a significantly higher cure rate than patients treated with praziquantel alone, with an OR = 1.84 (95% C.I. 1.01−3.36; p = 0.047). In *S. mansoni* infections, patients treated with combined therapy using artesunate and praziquantel also showed higher cure rates than patients treated with praziquantel alone, with OR = 2.81 (95% C.I. 1.09−7.24; p = 0.03). Similar higher cure rates were found in *S. japonicum* infection trials, with an overall OR = 1.92 (95% C.I. 0.34−10.74; p = 0.457). Subgroups difference using the Q test was no significant (p = 0.76). The summary odds ratio, regardless of the helminth species in question, was found to be OR = 2.07 (95% C.I. 1.27−3.36; p = 0.004), significantly favoring the combined therapy against praziquantel alone.

#### Artesunate combined with sulfadoxine-pyrimethamine vs. praziquantel

**Figure 4:**
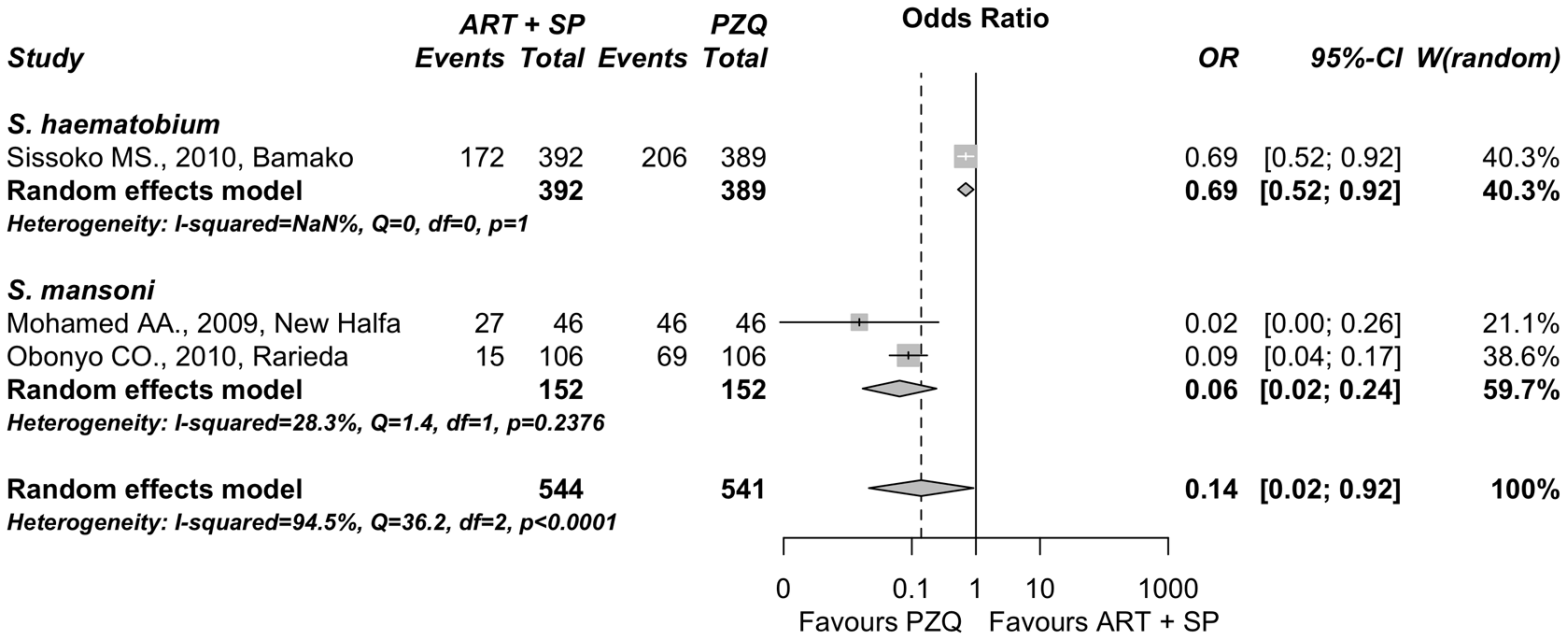
Forest plot of the random-effects subgroup meta-analysis comparing parasitological cure by artesunate *plus* sulfadoxine/pyrimethamine *vs.* praziquantel. Points represent odds ratios with their corresponding 95% C.I. Intermediate diamonds are combined odds ratios for each subgroup and the diamond at the bottom is the overall combined odds ratio. The vertical line emphasizes an odds ratio = 1 (no difference) and the dashed vertical line shows the value of the overall combined odds ratio. The original reports are labeled with author name, year and location (for details see table 3).

Three published reports (n = 1085) performed in Mali, Kenya and Sudan compared standard antimalarial treatment consisting on artesunate combined with sulfadoxine-pyrimethamine against praziquatel alone for schistosomiasis treatment [32], [33], [51]. Two of these reports referred to treatment for *S. mansoni* and one to treatment for *S. haematobium* infections (table 3). All studies that evaluated this approach including children that were followed up until 4 weeks post-treatment. We detected significant heterogeneity in the size of the effect among all the studies (Q = 36.2 (p<0.001), I^2^ = 94% (>50%)). Therefore, subgroup random-effects meta-analysis was performed (Figure 4). The cure rate for *S. haematobium* in the combined therapy trial is statistically lower compared with praziquantel alone: OR = 0.69 (95% C.I. 0.52−0.92; p = 0.01). Similary, for *S. mansoni* infections the combined therapy had significantly lower cure rates than those of praziquantel showing an overall OR of 0.06 (95% C.I. 0.02−0.24; p<0.001). Subgroups difference using the Q test was significat (p<0.001), as expected, taking into account that one OR was 0.69 for *S. haematobium* against 0.06 value for *S. mansoni*. Nevertheless, it could be valuable to report a summary effect of all the studies. Thus, regardless of the schitosome species in question, an overall odds ratio significantly less than one was found: OR = 0.14 (95% C.I. 0.02−0.92; p = 0.04). Therefore, the results do not appear to be in favor of the assayed antimalarial therapy as compared with praziquantel in schistosomiasis treatment.

#### Artesunate vs. placebo for the prevention of schistosomiasis

**Figure 5:**
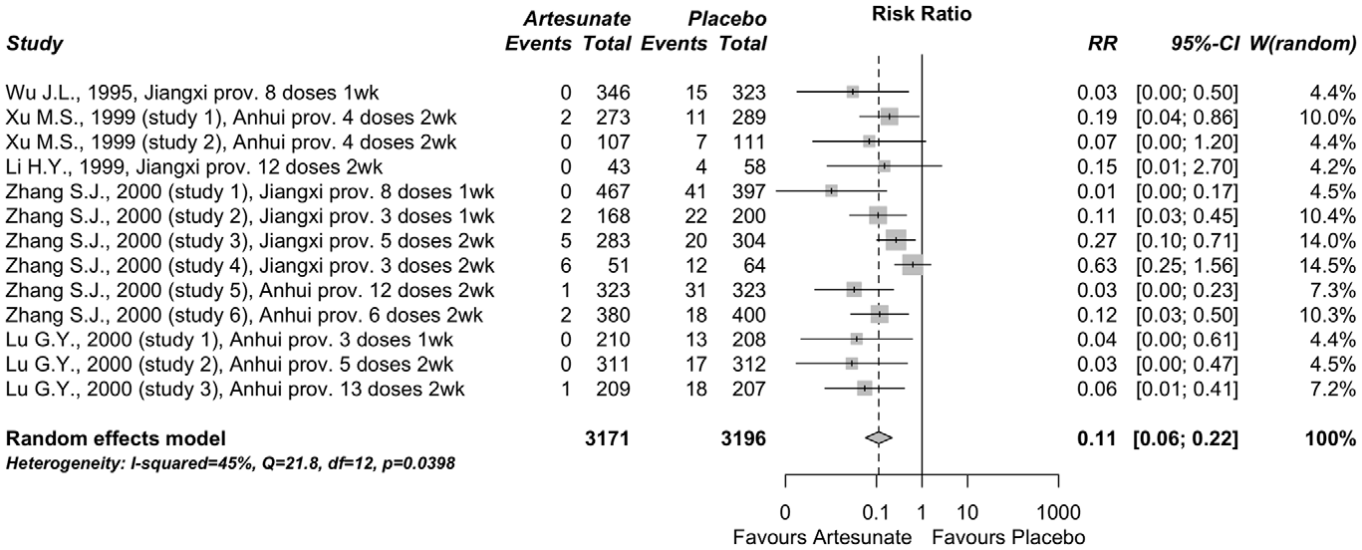
Meta-analysis comparing artesunate *vs*. placebo for chemoprophylaxis against schistosomiasis japonica. The mid-points of the lines represent the relative risk and the end-points of the lines show the corresponding 95% C.I. The diamond at the bottom is the overall relative risk. The vertical line emphasizes an relative risk = 1 (no difference) and the dashed vertical line shows the value of the overall relative risk. Relative risk <1 indicates a protective effect of artesunate. The original reports are labeled with author name, year, and location, number of dosis and interval of administration (for details see table 4).

A group of 5 published reports including 13 studies (n = 6367) were performed in different endemic areas of schistosomiasis in China investigating the efficacy of artesunate as a chemoprophylactic agent against *S. japonicum* infections [34]–[38]. Table 4 summarizes the key features, doses and outcomes of the studies included. We found significant heterogeneity among these studies (Q = 21.8 (p = 0.04), I^2^ = 45% (near 50%) and hence we used random-effects meta-analysis to estimate the prophylactic effect of artesunate (figure 5), which yielded a combined RR value of 0.11 (95% C.I. 0.06−0.22; p<0.001), pointing to a significant degree of schistosomiasis protection of artesunate in comparison with placebo.

#### Artemether vs. placebo for the prevention of schistosomiasis

**Figure 6:**
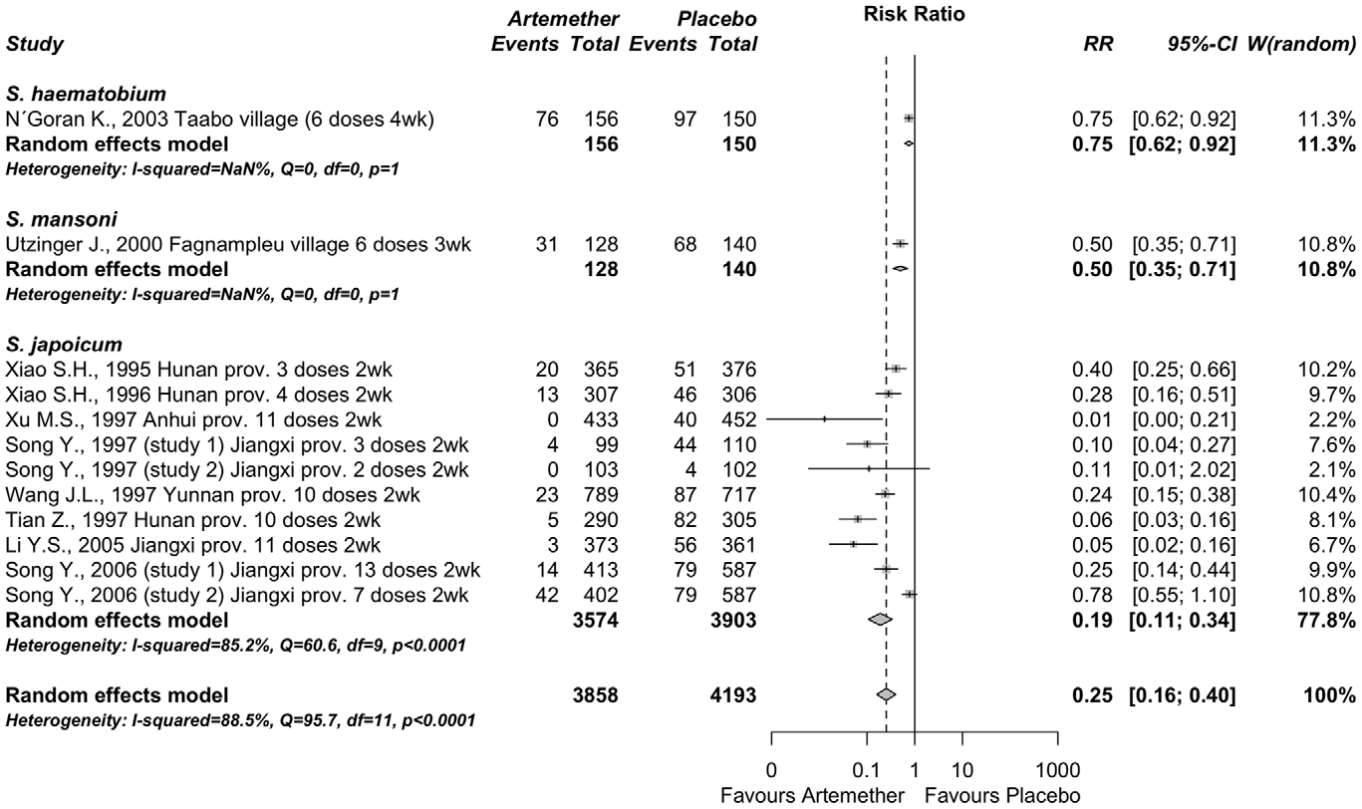
Subgroup meta-analysis comparing Artemether *vs*. placebo for chemoprophylaxis against schistosomiasis. The mid-points of the lines represent the relative risk and the end-points of the lines show the corresponding 95% C.I. Intermediate diamond symbols are combined relative risks for each subgroup and the diamond at the bottom is the overall combined relative risk. The vertical line emphasizes an relative risk = 1 (no difference) and the dashed vertical line shows the value of the overall relative risk. Relative risk <1 indicates a protective effect of artemether. The original reports are labeled as follows: author name, year, and location, number of dosis and interval of administration, for details see table 5.

A group of 10 published reports described 13 studies (n = 8051) in which it was assessed the efficacy of artemether for the prevention of schistosomiasis. Eight reports focused on *S. japonicum* infections in China [41]–[48] and 2 additional articles focused on *S. haematobium* [49] and *S. mansoni* [50] infections in Côte dIvore. The key features, doses and outcomes of the included studies were described in table 5. We detected significant heterogeneity among all the studies (Q = 95.7 (p<0.001), I^2^ = 89% (>50%)). Therefore, subgroup random-effects meta-analysis was performed to estimate the prophylactic effect of artemether (Figure 6). In *S. haematobium* and *S. mansoni* infections, a decrease in risk was observed: RR = 0.75 (95% C.I. 0.62–0.92; p = 0.006) and RR = 0.50 (95% C.I. 0.35–0.75; p<0.001), respectively, although these decreases are in fact significant more studies should be carried out because only one study of each parasite species is not sufficient. In addition, with *S. japonicum* infections we also found a significant prophylactic effect of artemether, with a RR = 0.19 (95% C.I. 0.11–0.34, p<0.001). Subgroups difference for the three parasites was significant by Q test (p<0.001), probably because the RR for *S. japonicum* is substantially lower than the other ones. The overall combined RR, regardless of the parasite species in question, was RR = 0.25 (95% C.I. 0.16–0.40, p<0.001), which is significantly in favor of schistosomiasis prevention by artemether.

#### Adverse effects

Regarding to artemisinin derivatives treatment, data for adverse events possibly related to artesunate in monotherapy compared with praziquantel were described in the 6 included publications in the present meta-analysis [25]–[30]. Those trials were not designed to evaluate differences in adverse events but some information can be extracted. Thus, the adverse effects were similary distributed between artesunate and praziquantel treatment and the most frequently adverse reactions were the incidence of gastric pain, headache, nausea and vomiting. With respect to the therapy with artesunate *plus* praziquantel *vs*. praziquantel alone, all the studies described that both drugs in combination were well tolerated and the most studies enphasized that the number of adverse effects were similar in both treatment regimens [25], [27], [29], [31]. In addition, none of studies included in this comparison revealed any additional side-effects caused by possible interactions between praziquantel and artemisinin derivatives. Interestingly, the three studies that evaluate the schistosomiaisis treatment using the standard antimalarial combination of artesunate *plus* sulfadoxine pyrimethamine *versus* praziquantel reported a significant lower incidence of side effects for the combinated therapy compared with praziquantel treatment [32], [33], [51].

Regarding the use of artemisinin derivatives as chemoprophylactic drug, artemether and artesunate were both well tolerated and no severe adverse events were recorded in the studies included in the meta-analysis. Only a few participants reported mild abdominal pain, headache, dizznes or slight fever, as other previous reviews mentioned [14], [52]. In addition, no significant changes in routine blood and urine tests, ECG, hepatic and renal functions before and after artemisinin derivatives administration [37].

#### Publication bias analysis

**Figure 7:**
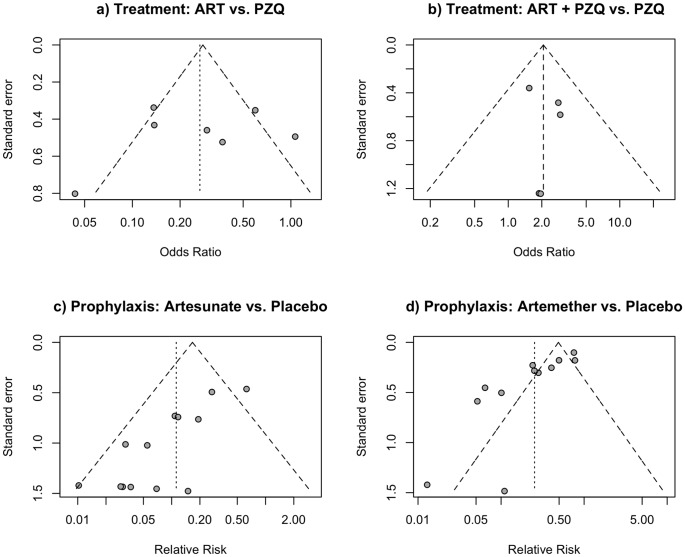
Funnel plots of differents subunits of analysis representing effect size against standard error. A vertical line indicates the estimate summary effect based on each particular model. A pseudo confidence interval region is drawn around this value with bounds equal to ±1.96 · SE, where SE is the standard error value from the vertical axis.

We assayed the possibility of publication bias evaluating the asymmetry of funnel plots (figure 7) and using the Egger’s regression test. We did not detect the presence of bias in the two meta-analysis of schistosomiasis treatment comparing artemisinin derivatives *vs.* praziquantel (Figure 1A) and artemisinin derivatives *plus* praziquantel *vs.* praziquantel alone (Figure 1B). Studies in both funnel plots show a symmetrical aspect. These visual findings were confirmed with the Egger’s test, which reported values of −0.38 (p = 0.7) and 0.49 (p = 0.7), respectively. However, presence of publication bias was detected in trials included in the two meta-analysis on schistosomiasis prophylaxis, the one related to artesunate *vs.* placebo (Figure 1C) and that of artemether *vs.* placebo (figure 1D). Both funnel plots show evidence of bias, more notable in the analysis including artemether. These visual asymmetries are confirmed by the Egger’s test that gave values of −5.29 (p<0.001) and −5.14 (p<0.001), respectively. We did not perform any assessment of publication bias in the meta-analysis focused on artesunate *plus* sulfadoxine-pyrimethamine *vs.* praziquatel due to low number of studies included in that sub-unit of analysis.

## Discussion

Schistosomiasis continues to be an important debilitating illness, in particular when associated with other potential causes of morbidity in tropical regions. Environmental changes, international travel and migratory populations increase the prevalence of schistosomiasis. The failure to develop an effective vaccine and the failure to eliminate snail populations mean that the control of schistosomiasis must rely on large-scale chemotherapy. The administration of praziquantel in a regimen of 40 mg/kg bodyweight in a single dose has been useful for controlling this disease in several countries such as Egypt, China, Brazil, the Philippines, Puerto Rico, Tunisia, Morocco, and Saudi Arabia [53]. However, rapid reinfection, incomplete cure rates, and evidence of praziquantel-tolerant schistosomes in the laboratory suggest a high risk of the development of praziquantel resistance [12].

Artemisinin derivatives currently offer the most important alternative for schistosomiasis treatment. They are effective against the juvenile stages of *S. mansoni*, *S. haematobium* and *S. japonicum*, but are less effective against adult worms. Our results demonstrate that artesunate in monotherapy does not offer an alternative for schistosomiasis treatment; none of the trials included in our meta-analysis seemed to significantly favor artesunate over praziquantel as monotherapy. Artesunate monotherapy may not be beneficial because its activity only affects the early stages of the parasite. In contrast, praziquantel acts against the mature forms of the parasite, curing 60 to 90 percent of patients. We measured efficacy of artemisinin derivatives at earliest after treatment in the context of continued disease transmission, in this sense some researchers have suggested that 3 weeks might be best [54]. However, more studies are needed to assess the effect of the evaluation period on the treatment outcome in those patients infected with schistosomiasis and treated with artemisinin derivatives.

Despite the incomplete efficacy of artemisinin derivatives alone in terms of cure rates, their pharmacological activities provide an excellent opportunity to combine with praziquantel. Our study confirms the Liu *et al.* [55] meta-analysis that support that artemisinin derivative *plus* praziquantel used in combination significantly increase the cure rates of schistosomiasis in comparison with praziquantel alone. The rationale for choosing combination treatments is to ensure rapid and reliable cures and to avoid the development of resistance to praziquantel. However, these estimated results cannot be regarded as definitive because they are based on diverse populations and the sample size of the trials included is small. Nevertheless, meta-analysis methodology may help to improve the power of small exploratory trials, including the broadest range of data (over multiple locations).

The adverse effects related with artemisinin derivatives were mild, artemether and artesunate were both well tolerated. Furthermore, studies that evaluated the antimalarial combination of artesunate plus sulfadoxine-pyrimethamine efficacy in schistosomiasis presented significant less adverse effects compared with praziquantel. Finally, no additional side-effects caused by possible interactions between praziquantel and artemisinin derivatives were detected across the studies. Taken together, these results indicate that the incorporation of artemisinin derivatives do not present any limitation related with the increase of adverse effects.

However, the incorporation of artemisinin derivatives in mass praziquantel administration has three important limitations: first, the cost-effectiveness implications; second, owing to the sub-optimal biopharmaceutical properties (very short half-life) of artemisinin derivatives repeated treatments are required [56], and third the use of artemisinin derivatives in mass administration could contribute to the emergence of artemisinin-resistant malaria [57]. Our meta-analysis shows that artesunate *plus* sulfadoxine-pyrimethamine does not offer a benefit over praziquantel-based therapy for *S. mansoni* and *S. haematobium* infections. Note that, to date no studies were carried out to evaluate changes on schistosomiasis endemicity as a function of the large-scale use of artemisinin based therapy in malaria control programmes.

Praziquantel is a poor choice for chemoprophylaxis because of its short half-life (1 to 1.5 hours) and because it cannot kill the schistosomula stage of the parasite [58]. However, a better option for schistosomiaisis prophylaxis could be the use of artemisinin derivatives because they are active against schistosomula stage [18]. In fact, our results have shown the prophylactic activity of artesunate and artemether. This is in accordance with some previous published reviews [13], [14], [52], [55] and a former meta-analysis performed by Wu *et al.* in 2003 [59]. Despite various levels of endemicity, different ecological settings and the diverse backgrounds of the participants, the prophylactic effect of artemisinin derivatives was demonstrated in each trial. It may be concluded that the prophylactic effect of artemisinin derivatives should be considered highly relevant in *S. japonicum* infection. Many clinical trials which focused on *S. japonicum* have shown that artesunate and artemether administered in multiple doses reduce the incidence of the infection to a significant extent, especially in those studies in which the target population was exposed to the infection at a specific moment because of flooding. However, the quality of the reports that evaluate the role of artemisinin derivatives as prophylatic drug was not optimal. In addition, we detected the presence of publication bias for artesunate and artemether prophylaxisis meta-analysis. Thus, the quantitative RR found might be affected in some extension by publication bias. New schistosomiasis trials focused on the prophylactic effect of artemisinin derivatives should be conducted paying attention to quality issues. In this sense, a previous report described the methodological limitations linked to clinical trials focused on schistosomiasis [60]. Finally, the meta-analysis of artemisinin derivatives focused on chemoprophylactic activity against schistosomiasis has also two key limitations: first, the difficult access to some trials published in Chinese language and second the lack of studies reporting efficacy of artemisinin derivatives as chemoprophylactic drug in *S. mansoni* and *S. haematobium* infections.

In sum, the combination of artemisinin derivatives with praziquantel seems to be the best option for the treatment of schistosomiasis, reflecting their complementary pharmacological profiles against this disease. In addition, the auxiliary benefit of artemisinin combination treatment administered to malaria patients should be evaluated in schistosome endemic areas. We also confirm the prophylactic effect of artemisinin derivatives across the different trials performed in China. Finally, we hope to provide clinicians and policy-makers with a convenient and evidence-based summary of the primary literature on which to base their decisions.

## Supporting Information

Table S1 **Summary characteristics and quality assessment of the published studies focused on schistosomiasis treatment.**
(DOC)

Table S2 **Summary characteristics and quality assessment of the published studies focused on schistosomiasis prophylaxis.**
(DOC)

Table S3 **PRISMA Checklist.**
(DOC)
