# Supplementary material for: Systematic Review and Meta-Analysis of Artemisinin Based Therapies for the Treatment and Prevention of Schistosomiasis
Source: PLoS One. 2012 Sep 21;7(9):e45867. doi: 10.1371/journal.pone.0045867 (PMC3448694; doi:10.1371/journal.pone.0045867)
Supplement: Table S1 — Summary characteristics and quality assessment of the published studies focused on schistosomiasis treatment. (DOC) [file pone.0045867.s001.doc]

Table S1: Summary characteristics and quality assessment of the published studies focused on schistosomiasis treatment

| ***Study and year of publication*** | ***Year (trial)*** | ***Parasite*** | ***Location (Country)*** | ***Population (age of participants)*** | ***Interventions*** | ***Follow up (weeks)*** | ***Generation of allocation sequence*** | ***Allocation concealment*** | ***Blinding*** | **A description of withdrawals or dropouts** | ***Study quality score ^1*^*** |
| --- | --- | --- | --- | --- | --- | --- | --- | --- | --- | --- | --- |
| Borrmann *et al.,* 2001 [25] | 2000 | *S. haematobium* | Moyen-Ogooué Province | Children (5-13) | PZQ + PBO | 8 | Adecuated | Adecuated | Double | Adecuated | 4 |
|  |  |  | (Gabon) |  | ART + PBO |  |  |  |  |  |  |
|  |  |  |  |  | ART + PZQ |  |  |  |  |  |  |
|  |  |  |  |  | PBO |  |  |  |  |  |  |
|  |  |  |  |  |  |  |  |  |  |  |  |
| De Clercq *et al.,* 2002 [26] | 2000 | *S. haematobium* | Lampsar village | Children (7-14) | ART | 5, 12, 24 | Unclear | Unclear | Unclear | Adecuated | 1 |
|  |  |  | (Senegal) |  | PZQ |  |  |  |  |  |  |
|  |  |  | Makhana village | Children (7-14) | ART | 5, 12, 24 | Unclear | Unclear | Unclear | Adecuated | 1 |
|  |  |  | (Senegal) |  | PZQ |  |  |  |  |  |  |
|  |  |  |  |  |  |  |  |  |  |  |  |
| Inyang-Etoh *et al.,* 2009 [27] | 2005 | *S. haematobium* | Adim village | Children (4-20) | PZQ + PBO | 8 | Unclear | Unclear | Unclear | Adecuated | 1 |
|  |  |  | (Nigeria) |  | ART + PBO |  |  |  |  |  |  |
|  |  |  |  |  | ART + PZQ |  |  |  |  |  |  |
|  |  |  |  |  | PZQ |  |  |  |  |  |  |
|  |  |  |  |  | ART |  |  |  |  |  |  |
|  |  |  |  |  | PBO |  |  |  |  |  |  |
|  |  |  |  |  |  |  |  |  |  |  |  |
| Keiser *et al.,* 2010 [28] | 2008 | *S. haematobium* | Guéssiguié | Children (8-16) | PZQ | 4 | Adecuated | Unclear | Open | Adecuated | 3 |
|  |  |  | (Côte d´Ivore) |  | Mefloquine |  |  |  |  |  |  |
|  |  |  |  |  | ART |  |  |  |  |  |  |
|  |  |  |  |  | ART-Mefloquine |  |  |  |  |  |  |
|  |  |  |  |  |  |  |  |  |  |  |  |
| De Clercq *et al.,* 2000 [29] | 1999 | *S. mansoni* | L. T. Salane village | Adults (n.d) | ART | 5, 12, 24 | Unclear | Unclear | Unclear | Adecuated | 1 |
|  |  |  | (Senegal) |  | PZQ |  |  |  |  |  |  |
|  |  |  |  |  | ART + PZQ |  |  |  |  |  |  |
|  |  |  |  |  |  |  |  |  |  |  |  |
| De Clercq *et al.,* 2000b [30] | 1998 | *S. mansoni* | Richard Toll village | Malaria (6-61) | ART | 5, 10 | Adecuated | Unclear | Unclear | Adecuated | 2 |
|  |  |  | (Senegal) | co-infection | PZQ |  |  |  |  |  |  |
|  |  |  |  |  |  |  |  |  |  |  |  |
| Hou *et al.,* 2008 [31] | 2003 | *S. japonicum* | Hunan Province | Residents (10-60) | PZQ+ART | 6 | Adecuated | Unclear | Double | Adecuated | 2 |
|  |  |  | (China) |  | PZQ |  |  |  |  |  |  |
|  |  |  |  |  |  |  |  |  |  |  |  |
| Sissoko *et al.,* 2009 [32] | 2007 | *S. haematobium* | Bamako | Children (6-15) | ART+SP | 4 | Adecuated | Adecuated | Double | Adecuated | 4 |
|  |  |  | (Mali) |  | PZQ |  |  |  |  |  |  |
|  |  |  |  |  |  |  |  |  |  |  |  |
| Mohamed *et al.,* 2009 [52] | 2008 | *S. mansoni* | New Halfa | Children (8-17) | ART+SP | 4 | Unclear | Unclear | Open | Adecuated | 2 |
|  |  |  | (Sudan) |  | PZQ |  |  |  |  |  |  |
|  |  |  |  |  |  |  |  |  |  |  |  |
| Obonyo *et al.,* 2010 [33] | 2009 | *S. mansoni* | Rarieda | Children (6-15) | ART+ SP | 4 | Adecuated | Adecuated | Open | Adecuated | 4 |
|  |  |  | (Kenya) |  | PZQ |  |  |  |  |  |  |

***Score ^1*^:*** Range 0~4 according to modified Jadad score
