# Supplementary material for: Systematic Review and Meta-Analysis of Artemisinin Based Therapies for the Treatment and Prevention of Schistosomiasis
Source: PLoS One. 2012 Sep 21;7(9):e45867. doi: 10.1371/journal.pone.0045867 (PMC3448694; doi:10.1371/journal.pone.0045867)
Supplement: Table S2 — Summary characteristics and quality assessment of the published studies focused on schistosomiasis prophylaxis. (DOC) [file pone.0045867.s002.doc]

Table S2: Summary characteristics and quality assessment of the published studies focused on schistosomiasis prophylaxis

| ***Study and year of publication*** | ***Year (trial)*** | ***Parasite*** | ***Location (Country)*** | ***Population (age of participants)*** | ***Interventions*** | ***Follow up (weeks)*** | ***Generation of allocation sequence*** | ***Allocation concealment*** | ***Blinding*** | **A description of withdrawals or dropouts** | ***Study quality score^1*^*** |
| --- | --- | --- | --- | --- | --- | --- | --- | --- | --- | --- | --- |
| Wu *et al.,* 1995 [34] | 1993 | *S. japonicum* | Jiangxi province (China) | Residents (5-60) | Artesunate | 4 | Unclear | Unclear | Double | Adecuated | 2 |
|  |  |  |  |  |  |  |  |  |  |  |  |
| Xu *et al.,* 1999 [35] | 1997 | *S. japonicum* | Jiangxi province (China) | Residents (6-65) | Artesunate | 4 | Unclear | Unclear | Unclear | Unclear | 0 |
|  |  |  |  |  |  |  |  |  |  |  |  |
| Li *et al.,* 1999 [36] | 1997 | *S. japonicum* | Jiangxi province (China) | Residents (5-60) | Artesunate | n.a. | Unclear | Unclear | Unclear | Unclear | 0 |
|  |  |  |  |  |  |  |  |  |  |  |  |
| Zhang *et al.,* 2000 [37] | 1993 | *S. japonicum* | Jiangxi, Hubei, Anhui province (China) | Residents (6-65) | Artesunate | 4 | Unclear | Unclear | Double | Unclear | 0 |
| Lu *et al.,* 2000 [38] | 1999 | *S. japonicum* | Anhui province | Residents (5-60) | Artesunate | 4 | Unclear | Adecuated | Unclear | Unclear | 1 |
|  |  |  |  |  |  |  |  |  |  |  |  |
| Goran *et al.,* 2001 [49] | *2000* | *S. haematobium* | Taabo village (Côte d´Ivore) | Children (5-15) | Artemether | 3 | Adecuated | Adecuated | Double | Adecuated | 3 |
|  |  |  |  |  |  |  |  |  |  |  |  |
| Utzinger *et al.,* 2000 [50] | 1998 | *S. mansoni* | Fagnampleu village (Côte d´Ivore) | Schoolchildren (n.d.) | Artemether | 3 | Adecuated | Unclear | Double | Adecuated | 2 |
| Xiao *et al.,* 1995 [41] | 1994 | *S. japonicum* | Hunan province (China) | Residents (4-65) | Artemether | 4 | Adecuated | Adecuated | Double | Adecuated | 3 |
|  |  |  |  |  |  |  |  |  |  |  |  |
| Xiao *et al.,* 1996 [46] | 1995 | *S. japonicum* | Yunnan province (China) | Residents (4-65) | Artemether | 4 | Adecuated | Adecuated | Double | Unclear | 3 |
|  |  |  |  |  |  |  |  |  |  |  |  |
| Xu et al*.,* 1997 [43] | 1996 | *S. japonicum* | Anhui province (China) | Residents (6-65) | Artemether | 4 | Unclear | Adecuated | Double | Adecuated | 3 |
|  |  |  |  |  |  |  |  |  |  |  |  |
| Tian et al*.,* 1997 [44] | 1996 | *S. japonicum* | Hunan province (China) | Residents (5-60) | Artemether | 4 | Unclear | Adecuated | Double | Adecuated | 3 |
|  |  |  |  |  |  |  |  |  |  |  |  |
| Song *et al.,* 1998 [45] | 1996 | *S. japonicum* | Jiangxi province (China) | Flood workers (18-40) | Artemether | 5 | Unclear | Unclear | Unclear | Adecuated | 1 |
| Wang *et al.,* 1997 [46] | 1993 | *S. japonicum* | Yunnan province (China) | Residents (3-60) | Artemether | 4 | Unclear | Unclear | Unclear | Adecuated | 1 |
|  |  |  |  |  |  |  |  |  |  |  |  |
| Li *et al.,* 2005 [48] | 2004 | *S. japonicum* | Jiangxi province (China) | Residents (6-60) | Artemether | 4 | Unclear | Unclear | Double | Adecuated | 2 |
|  |  |  |  |  |  |  |  |  |  |  |  |
| Song *et al.,* 2006 [47] | 2004 | *S. japonicum* | Jiangxi province (China) | Residents (6-65) | Artemether | 6-8 | Unclear | Unclear | Double | Adecuated | 2 |

***Score ^1*^:*** Range 0~4 according to modified Jadad score; n.d. data not described
